# A Wearable Textile 3D Gesture Recognition Sensor Based on Screen-Printing Technology

**DOI:** 10.3390/s19235068

**Published:** 2019-11-20

**Authors:** Josue Ferri, Raúl Llinares Llopis, Jorge Moreno, Javier Ibañez Civera, Eduardo Garcia-Breijo

**Affiliations:** 1 Textile Research Institute (AITEX), 03801 Alicante, Spain; josue.ferri@aitex.es (J.F.); jmoreno@aitex.es (J.M.); 2 Departamento de Comunicaciones, Universitat Politècnica de València, 03801 Alcoy, Spain; rllinares@dcom.upv.es; 3 Instituto Interuniversitario de Investigación de Reconocimiento Molecular y Desarrollo Tecnológico (IDM), Universitat Politècnica de València, Universitat de València, 46022 Valencia, Spain; jibanyez@eln.upv.es

**Keywords:** gesture recognition, screen-printing, 3D touchpad, e-field sensors, wearables, touchless

## Abstract

Research has developed various solutions in order for computers to recognize hand gestures in the context of human machine interface (HMI). The design of a successful hand gesture recognition system must address functionality and usability. The gesture recognition market has evolved from touchpads to touchless sensors, which do not need direct contact. Their application in textiles ranges from the field of medical environments to smart home applications and the automotive industry. In this paper, a textile capacitive touchless sensor has been developed by using screen-printing technology. Two different designs were developed to obtain the best configuration, obtaining good results in both cases. Finally, as a real application, a complete solution of the sensor with wireless communications is presented to be used as an interface for a mobile phone.

## 1. Introduction

The ability of computers to recognize hand gestures is essential for progress in human-computer interaction (HCI) and human machine interaction (HMI) [1]. Gesture recognition enables humans to interact with computers and machines without any physical contact in a more natural way. It is an alternative method for interacting with computers without using traditional peripheral devices, such as the keyboard or the mouse. One of the most relevant devices is the Microsoft Kinect sensor, now updated as Azure Kinect DK sensor, both consisting of advanced sensing hardware combining a VGA (Video Graphics Array) video camera, an infrared depth sensor and a multi-array microphone allowing people to interact with the games using their body [2]. In addition, this device includes a complex microprocessor that is able to run advanced machine-learning algorithms on a graphics processing unit (GPU) in parallel, tracking simultaneously up to six people. Although face and other body parts, considered as full body interaction [3], can be used to interact with computers, hand gesture recognition is the most popular solution for different reasons. Factors such as end-user application, reliability, cost, and context influence the choice of one technology in detriment of another.

Hand gesture recognition based human machine interface (HMI) is an attractive topic because it is especially important in both industrial and domestic applications, to evolve the way we interact with our environment. Hand gesture recognition allows us to control elements in a 3D space, rather than traditional interfaces, such as the mouse or the keyboard, that are limited to a 2D environment. These features have an important impact on CAD (Computer-Aided Design) applications, 3D gaming, or Virtual Reality. Hence, gesture recognition has a huge application ranging from very simple applications to interact with home appliances such as the TV [4] to complex systems of telemedicine [5]. In addition, hand gesture recognition has a promising future in some circumstances where hands are not able to touch equipment, such as in medical environments [6], helping impaired people to communicate [7], game-based rehabilitation applications [8,9], cooking scenarios [10], controlled robots at industrial environments [11], vehicle interfaces [12,13], or military needs [14].

The design of a successful hand gesture recognition system must address two main characteristics: functionality and usability [15]. Functionality refers to the set of functions, gestures, or services that the system offers to the users, whereas usability refers to the performance and user experience to perform specific purposes efficiently. Generally, the video technology is used for general motion recognition and has also been used for hand gesture recognition. Most of the systems based on video technology, although able to detect many gestures, are faced with other challenges, such as lighting variation, hand location, orientation, or the changes in the background. Nevertheless, this technology has promising results as soon as the complex algorithms needed for real time applications are able to be embedded on hardware such as FPGA (Field-Programmable Gate Array) or ASIC (Application-Specific Integrated Circuit) devices. As a drawback, the powerful microprocessors required raise the price of the final product. Another factor to consider is the invasion of privacy inherent in video-based recognition.

As an alternative, there exist wearable devices for user interaction with bending sensors [16,17], LEDs (Light-Emitting Diode) [18], electrical impedance sensors on the skin [19], and accelerometers to be worn on gloves. These alternatives have shown good results with high classification accuracy but also some disadvantages such as the calibration of the orientation or the need to use gloves that may be uncomfortable for the user. 

Another group of solutions are based on proximity sensors that have limited gesture recognition but good accuracy and low price. These sensors use different technologies depending on the way they process the signals involved. Sensors based on radar signals [20,21,22] make use of transmission and reflection of RF (Radio Frequency) waves, determining the time delay between transmitted and received waveforms. Other sensors, sound sensors, apply similar techniques but working in different frequencies [23,24]. These solutions use sonar systems that transmit inaudible sound signals and track the echoes of the hand with its microphones. 

Analysing commercial products and the evolution of the gesture recognition market, there is a natural evolution from the touchpads [25,26,27], where contact with sensors is needed [28], to touchless sensors [29]. Regarding touchpad technologies, two main types of sensors can be found: capacitive and resistive [30]. Of the two, only capacitive sensors can be used as touchless detecting hand gestures. In this field, there exist different approaches, but all of them have presented good results in low power consumption, seamless integration, and low cost [31].

Focusing on hand gesture recognition with capacitive touchless sensors [32,33], the literature presents many approaches. Some authors have classified the technologies as active or passive. Other authors have found up to four different modes of operation [34]. Each one has benefits and limitations but, despite the limitations, capacitive sensors have been shown to be able to sense human activities. Moreover, one of the advantages of this technology is that it is possible to use capacitive sensors on non-rigid substrates, supporting flexible and stretchable substrates [35,36]. All these features offer an opportunity to overcome challenges that smart textiles demand [37] as user interfaces embedded into textiles and fabrics [38]. Applications such as a sofa pillow that integrates a smart TV remote control, an intuitive embedded interface to activate a recliner armchair, or smart cloths that are able to interact with external computers are examples that could take advantage of this innovation [39].

This paper shows the behaviour and influence of different smart textile materials used as capacitive sensors for the purpose of hand gesture recognition. The electrodes that conform the sensor structure are printed on textiles substrates using screen printing technology. All the study uses is the 3D GestIC^®^ sensor from Microchip Technology Inc (Chandler, Arizona, USA) with their MGC3130 processor. The different smart textiles prototypes presented are compared with the reference sensor that fits with the design recommendations of Microchip. The research is divided in two different parts, one corresponding to the design and working principles and the other one focusing on the development of the system and the obtained results. Two three-conductive layers designs are presented. Both of them present five reception electrodes and one transmission electrode. The studied parameters are the conductivity and relative permittivity for the different used inks as well as the thickness and relative permittivity of the fabrics. Moreover, the real capacitance values obtained for each individual electrode are presented and compared with the theoretical ones. The research also presents the sensitivity of each individual development and the results detecting gestures. Finally, a real-world application is presented, a mouse for a mobile phone.

## 2. Design and Working Principle

This section presents the design of the proposed system based on a 3D GestIC^®^ sensor from Microchip Technology Inc. The 3D GestIC^®^ sensor is a combination of a gesture Microchip controller and a set of sensor electrodes. Microchip’s GestIC^®^ is a 3D sensor technology which utilizes an electric field for advanced proximity sensing. Usually this sensor is made by using a PCB (Printed Circuit Board) technology on a rigid or flexible substrate normally of polymer materials [40].

### 2.1. Working Principle

**Figure 1:**
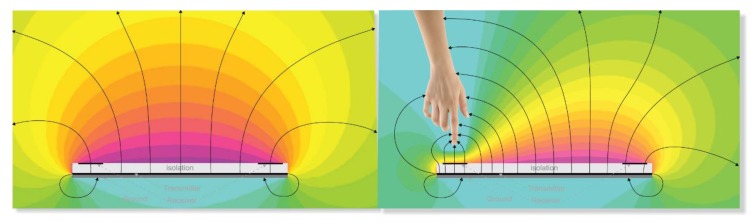
Field lines generated by the transmission Tx electrode. The reception Rx electrodes are located inside of the generated field. On the left side of the Figure, the field lines are shown when they are not modified by any conductor object. On the right side of the Figure, a hand is causing a modification of the field lines, leading to a variation in the signal received by the Rx electrodes. Source: Microchip Technology Inc.

The functioning of the sensor is based on the modification, in this case due to the proximity of hands, of the lines of an electric field. The variation of the distribution of the lines of the electric field is detected by a controller and interpreted by means of an algorithm, into different types of gestures that can be visualized conveniently. To generate a spatial electric field, i.e., in three dimensions, an electrode acting as an antenna is utilized. This electrode conducts an alternating signal and is usually named transmission electrode or Tx electrode. The behaviour of the non-radiative near field (reactive) of the electromagnetic fields dominates close to the transmitter. Using an electrode with a geometry much smaller than the wavelength and working close to the electrode, the magnetic component of the generated field is insignificant and there is no wave propagation. The electric component is quasi-static, enabling to detect possible conducting elements modifying the mentioned field with hands, fingers, etc. That is, if a person places their hands inside of the emitting area, a perturbation of the lines of the electric fields is created, due to the deviation of the lines to ground using the intrinsic conductivity of the human body. Using a series of reception electrodes, Rx electrodes, it is possible to determine the value and position in the electric field (Figure 1).

**Figure 2:**
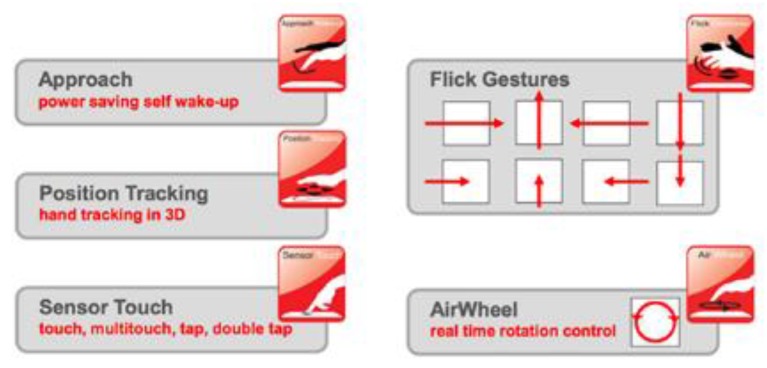
Gestures recognized by the internal algorithm of MGC3130: approach detection, position tracking in 3D, sensor touch (touch, multitouch, tap, and double tap), flick gestures, circle gestures, and airwheel. Source: Microchip Technology Inc.

Microchip Technology’s MGC3130 is a three-dimensional (3D) gesture recognition, motion tracking and approach detection controller based on Microchip’s patented GestIC^®^ technology for embedded usage. It enables user command input with natural hand and finger movements [41,42]. MGC3130 is able to generate a Tx signal of about 100 kHz, that corresponds to a wavelength of 3 km and has the capacity to acquire signals from 4 or 5 Rx electrodes. Using a sensor with electrodes in the 4 cardinal points and an electrode in the centre, this integrated circuit is able to recognize the variations and position of the perturbations of field that a hand produces in the delimited area over the sensor. The maximum range of detection is 15 cm in the perpendicular axis to the sensor. The central electrode is usually used as a touchpad and can be removed in case the sensor is only used to detect gestures. As aforementioned, the MGC3130 controller utilizes an algorithm to detect the following gestures: approach detection, position tracking in 3D, sensor touch (touch, multitouch, tap and double tap), flick gestures, circle gestures, and airwheel (Figure 2).

**Figure 3:**
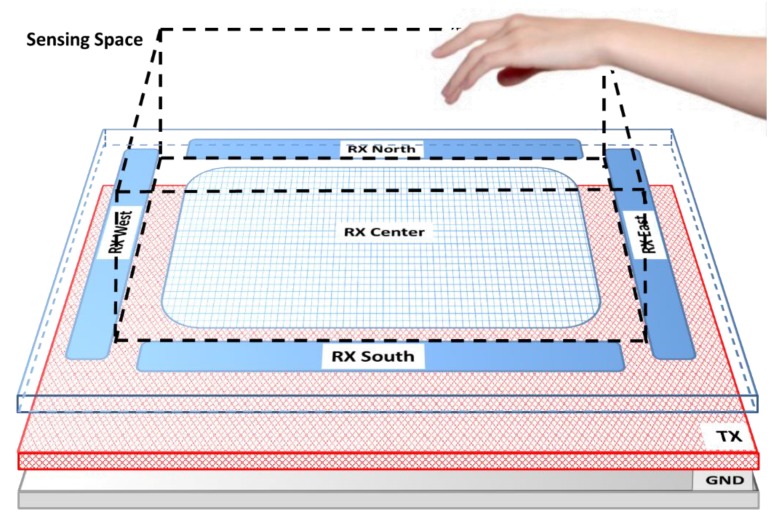
Standard sensor used by Microchip. It consists of a first layer where four Rx electrodes are located on each of the cardinal points as well as a central Rx electrode. This layer is separated from the bottom layer that contains the Tx electrode by a dielectric. The ground plane layer is optional and would be located below the Tx electrode layer. The sensitive area is just delimited by the four perimeter Rx sensors. Source: Microchip Technology Inc.

The design suggested by Microchip [40] consists of a sensor made in rigid or double-sided flexible PCB (Printed Circuit Board), where the Rx electrodes are distributed on the four cardinal points of the upper face and on the centre. The Tx electrode is located on the underside. This design is used when the operation does not need a battery, and the electrical noise is low. When the external noise is high or the functioning uses a battery, three layers are needed due to the addition of a ground plane. In the PCB designs, four conductive layers are implemented, leaving one of them with no use. In the case of utilizing a four layer design, the upper layer is used for Rx electrodes, the second internal layer for the Tx signal and the underside layer for the ground plane (Figure 3).

The Rx and Tx layers must be of conductive material such as copper but can be of indium-tin oxide (ITO) or similar. The electrode isolation can be made of any non-conductive material (FR4, glass, PET, etc.). Microchip proposes two designs [40] for MGC3130, of different and compatible sensors:

1. Standard sensor (Tx signal amplitude of 2.85 V). Useful for small or medium-sized devices and mandatory for devices with a weak connection to ground, that is, with battery.
2. Booster sensor (Tx signal amplitude between 5 and 18 V) allowing bigger sensors and recognition ranges.

**Figure 4:**
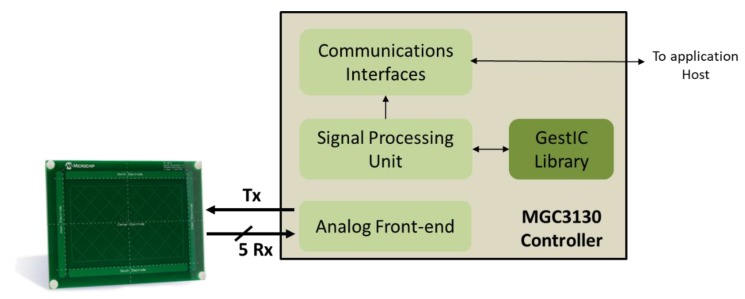
MGC3130 Block Diagram, composed of an analog front-end module that allows to generate the transmission Tx signal and receive the signals from the 5 Rx electrodes. The signals, properly processed, are transferred to the Signal Processing Unit that, together with the GestIC library, processes and converts them into the different programmed gestures. Lastly, there is a communication block with a host. Source: Microchip Technology Inc.

Figure 4 shows the block diagram of the MGC3130 controller. It consists of the analogue front-end unit connected to the Tx and Rx electrodes, the signal processing unit that receives data from the front-end and is assisted by a GestIC library and, finally, a communication interface for the data interconnection between MGC3130 and another microcontroller.

**Figure 5:**
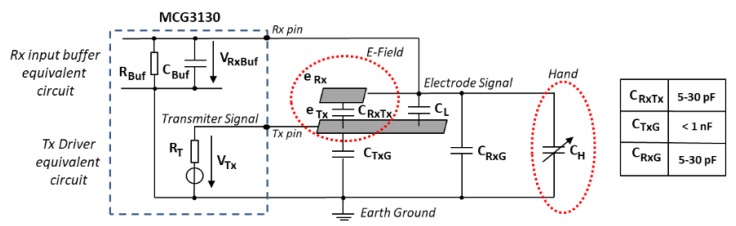
Equivalent simplified circuit of the combination sensor-MGC3130. Source: Microchip Technology Inc.

The MGC3130 controller is parameterizable, that is, it can be reconfigured for each type of sensor and application. Thus, the most important part of the design of this system is the sensor characterization. It can be used to detect hand movements or only finger movements, influencing in the size of the sensor. This research uses the hand, which can be used in parallel, perpendicularly or with a certain angle to the sensitive area. The sensitive area is always defined inside of the four cardinal Rx electrodes. The geometrical form of the sensitive area is a key factor. This depends on the electrode geometry but also on the electrode location. For the design of the sensor, it is necessary to have some knowledge of its electrical parameters (shown in Figure 5). This electrical model allows us to evaluate the characteristics of the system and points out the dependencies between the electrodes, the MGC3130 controller and the hand. In particular, the capacitances of the electrodes which are coupled to the input/output sections of the MGC3130 integrated circuit. 

The output signal, V_Tx_, is found on the Tx pin of the MGC3130 integrated circuit, whose capacitance to ground is C_TxG_. e_Tx_ and e_Rx_ represent the transmission and reception electrodes. The parameterization of the corresponding circuit is based on the C_RxTx_, C_RxG_, and C_L_ capacitances. C_RxTx_ is the capacitance between the Tx and Rx electrodes, C_RxG_ is the capacitance between the Rx electrode and ground and C_L_ is the coupled capacitance between the transmission Tx pin and the Rx reception line. Finally, C_H_ is the capacitance between the hand and the Rx electrode. C_H_ is represented as a variable capacitor, since the corresponding capacitance depends on the hand and its position regarding the Rx electrodes. The e_Rx_ reception electrode measures the electric field potential. When a conductive object, like a hand, interacts with the electric field, the C_H_ capacitance of the reception electrode changes in the range of femtofarads and the corresponding variation is detected by the MGC3130 integrated circuit. The embedded table in Figure 5 shows the typical range of these capacitances.

### 2.2. Microchip Sensor Design 

The sensor can have different sizes and shapes, such as square, circular, oval, or rectangular, as long as the 1:3 length to width ratio is not exceeded. Microchip recommends a maximum of 140 × 140 mm (or a 140 mm diameter) and a minimum of 20 × 20 mm (diameter of 20 mm). The sensor must have a minimum of two layers but can be made with three layers: Rx on the upper layer, Tx on the bottom layer and, if necessary, GND as a third layer [40].

**Figure 6:**
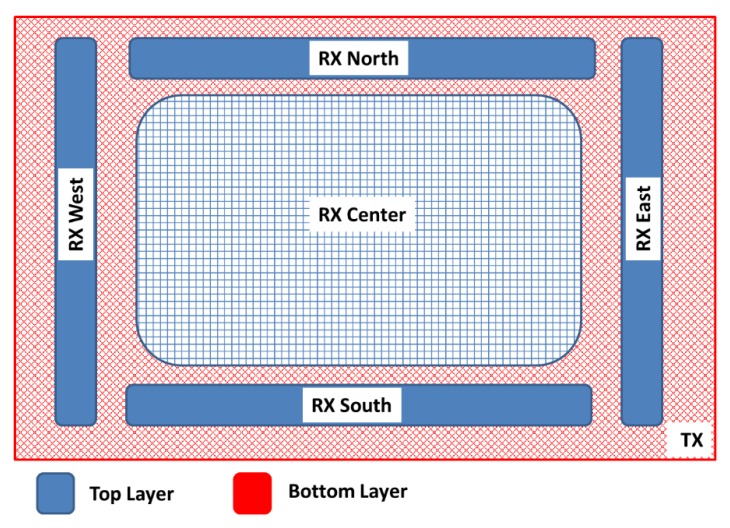
Basic scheme of the gesture sensor recommended by Microchip. Source: Microchip Technology Inc.

Figure 6 shows a typical design. On the upper layer, the four perimeter electrodes can be observed, as well as the optional central electrode, whereas the Tx electrode is located on the bottom layer. The size of the Rx electrodes determines the sensitive area. These Rx electrodes must always remain delimiting the aforementioned sensitive area to obtain the maximum resolution for the x, y, and z coordinates.

**Figure 7:**
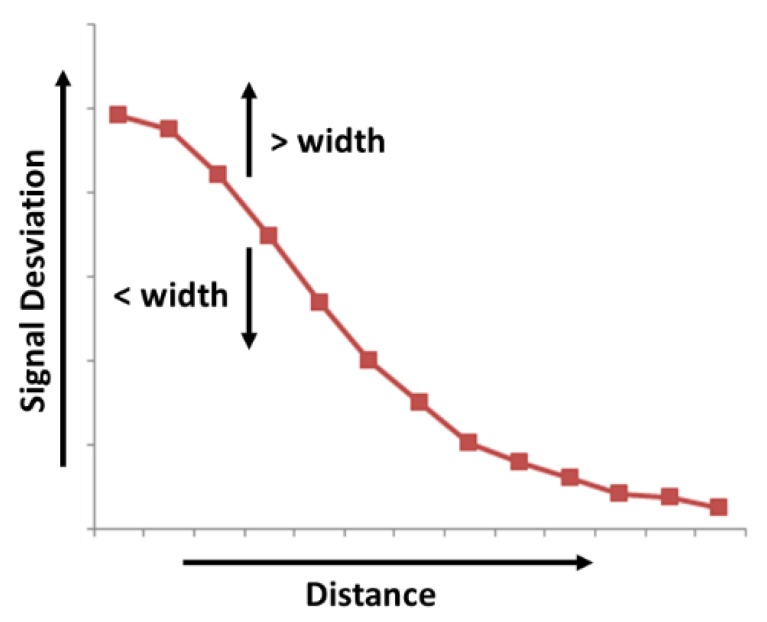
Variation of signal deviation received by Rx in function of the distance of the hand to the sensor and of the Rx electrode width.

With the aim of obtaining a high sensitivity in the sensitive area, the area of the reception electrodes and the hand should be of the same order of magnitude. Hence, the four Rx electrodes of the cardinal points should have a lengthened shape to increment the coupling between the Rx electrodes and the hand. If the recognition range must be symmetric in both directions, the electrode design must be symmetric. In any case, the length of both vertical and horizontal electrodes must be balanced. The recommended distance between the Rx electrodes is of 1 to 5 mm. The most influential factor of the sensitivity to the hand is the width of the perimeter Rx electrodes. The bigger the width of the electrode is, the higher the sensitivity is. The receiver signal sensitivity measures the influence of the hand capacitance when a hand approaches the system. As a consequence, the Rx signal changes in presence of a hand, deviating from a reference value. The value of the deviation is measured and named Signal Deviation. It is the basis for the recognition of gestures [41]. Microchip references the signal deviation obtained in MGC3130 with respect to the distance of the hand compared with the width of the Rx electrodes (Figure 7). Logically, the larger the distance of the hand on the sensitive area is, the less the signal deviation is. The width of the electrode also influences the signal deviation. The wider the Rx electrode is, the greater the signal deviation is. However, an increase in the area of the Rx electrode implies a limitation in the range of gesture recognition. A best practice is to choose an Rx electrode width between 4% and 7% of the length. Moreover, an overly large Rx electrode causes higher capacitance between Rx and Tx and also between Rx and the ground. As a result of that, a loss of signal sensitivity is produced.

In the case of the central Rx electrode, a meshed design is recommended. The mesh design increases the sensitivity due to the reduction of the effective area of the Rx electrode and to the coupling to the Tx electrode. An acceptable value for the mesh would be around 5% and 20%.

The C_RxGND_ capacitance must be as low as possible. That is, the distance between the Rx electrodes and ground must be as high as possible.

Regarding the Tx electrode, it must be located under the Rx electrodes and must have a shape and size to cover all the reception area. On the other hand, the Tx electrode must have a low coupling with the Rx electrodes (C_RxTx_) and with ground (C_TxG_). To reduce the C_RxTx_ capacitance, the distance between the Tx and the Rx electrodes must be as long as possible. The optimum distance between these two layers (t) will depend on the relative permittivity (*ε_r_*) of the isolation material between both layers. Microchip recommends a t > *ε_r_*/5, hence, for a FR4 material (glass-reinforced epoxy laminate material *ε_r_* = 5), the thickness can be of 1 mm, for plastic (*ε_r_* = 3), it can be of 0.6 mm and, for glass (*ε_r_* = 6), it can be of 1.2 mm. Microchip recommends a minimum value between 1 to 2.5 mm, a value of 50% to 100% of the Rx electrode width being considered optimal. In any case, the calculated thickness will be the minimum one, achieving a better response as this distance increases.

**Figure 8:**
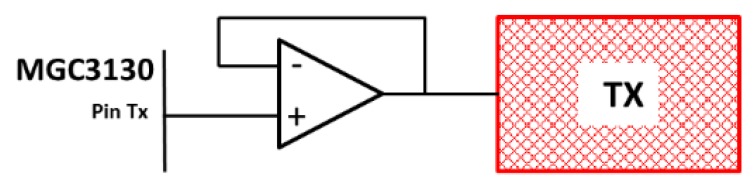
An op-amp buffer must be inserted between the Tx pin and the Tx electrode in case C_TxGND_ is greater than 1 nF. Source: Microchip Technology Inc.

**Figure 9:**
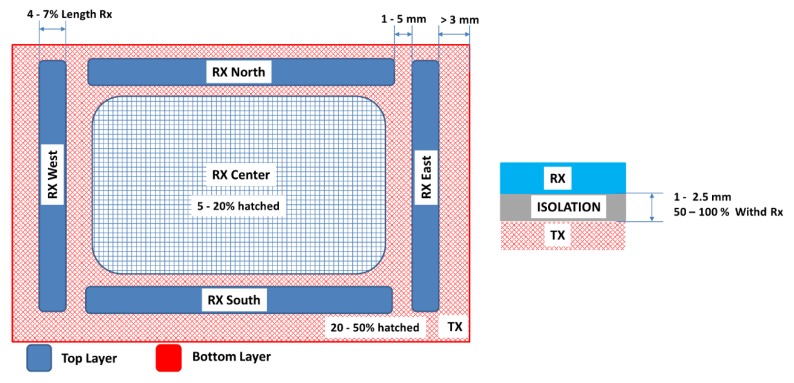
Basic design parameters recommended by Microchip. Source: Microchip Technology Inc.

Regarding the C_TxGND_ capacitance, it cannot be greater than the conductive capacitance of MGC3130 that is 1 nF. To reduce it, the Tx electrode must have a large surface and a meshed design of between 20% and 50% of the surface. When the C_TxGND_ capacitance is not low enough, an operational amplifier (op-amp) must be inserted between the Tx pin and the Tx electrode (Figure 8). Lastly, Microchip recommends an overlap between the Tx electrode and the external edges of the Rx electrodes greater than 3 mm (Figure 9).

Apart from the Rx and Tx layers, a third layer, GND, must be added in case of systems with no battery in high noise environments or in case of battery operation. When the system operates with batteries, this GND layer is mandatory but, in systems with ground connection, this layer is optional. In any case, the GND layer confers stability and noise immunity but at the expense of losing sensitivity, between 10% and 20%. As aforementioned, the C_TxGND_ capacitance must be lower than 1 nF. To achieve this, the thickness between the Tx and GND layers must be increased using materials with a greater relative permittivity and mesh design.

The Rx and Tx electrodes are not only limited to the sensitive area of the sensor but are also formed by the conductive lines that join these electrodes with the MGC3130 controller. Hence, these conductive lines influence the gesture detection as well. For this reason, these lines must be designed with as shorter length as possible, inside of the sensitive area and, if possible, with a distance from the Tx electrode larger than 0.15 mm. 

**Figure 10:**
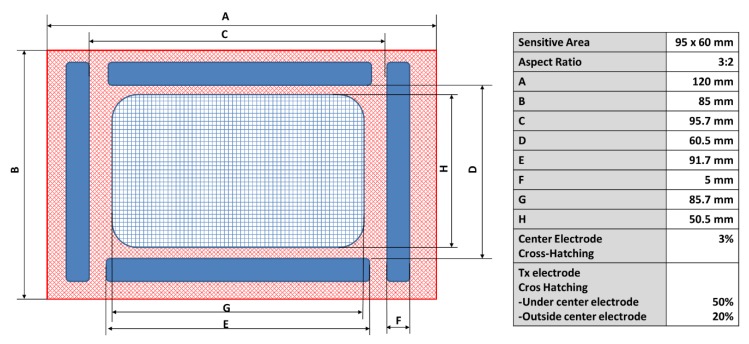
General characteristics of the 95 × 60 sensor from Microchip. Source: Microchip Technology Inc.

Before proceeding with the design of the sensor with a textile, a study of the sensor of Microchip was performed. The reference sensor had a sensitive area of 95 × 60 mm with a size of 120 × 85 mm. This study helped with the validation of the results obtained with the textile version. The sensor characteristics are shown in Figure 10.

**Figure 11:**
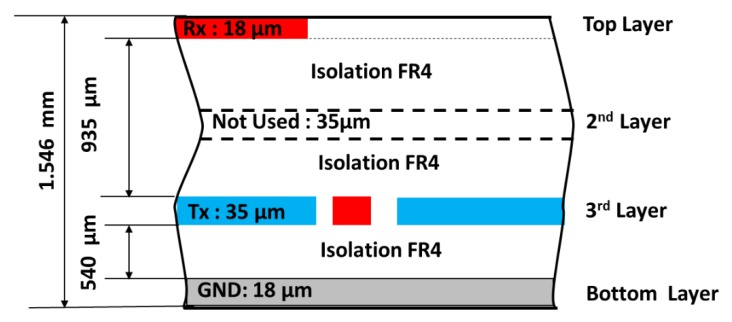
Crosscut of the PCB of the 95 × 60 sensor; dimensions and number of layers. Source: Microchip Technology Inc.

The PCB (Printed Circuit Board) follows a four layers design. The Rx electrodes are located on the upper layer, the second layer (internal) is not used, the third layer (internal) contains the Tx electrode, and the bottom layer includes the GND plane. The dielectric between the conductive layers is of FR4 material. The cross distribution of the PCB is shown in Figure 11. The relative permittivity (*ε_r_*) of FR4 is considered to be 4.8.

Table 1 shows the different values obtained for C_TxRx_. The range acceptable by Microchip for this capacitance oscillates between 5 and 30 pF. The value for this PCB provided by Microchip is shown as well. Note the higher value corresponding to the central electrode due to its larger area.

Table 2 shows the different values obtained for C_TxGND_. The value for this capacitance acceptable by Microchip is lower than 1 nF. 

Table 3 shows the different values obtained for C_RxGND_. The range for this capacitance acceptable by Microchip oscillates between 5 and 30 pF.

**Figure 12:**
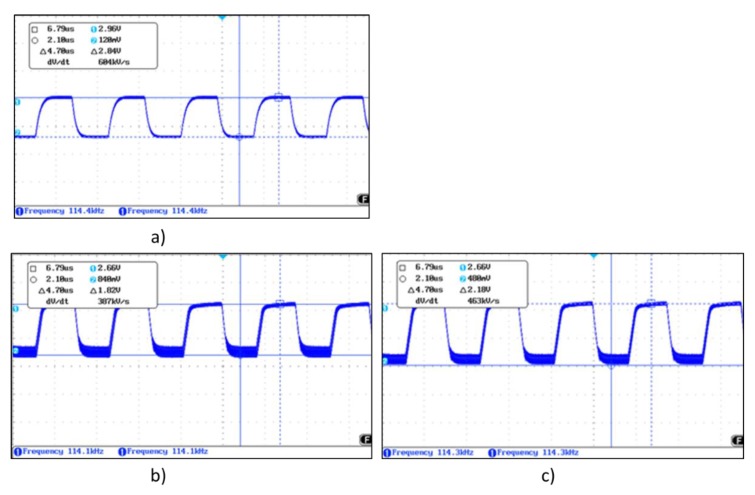
(**a**) Waveform of the transmission signal, (**b**) the received Rx signal with no object modifying the field lines, (**c**) the received Rx signal with an object modifying the field lines.

Figure 12a shows the Tx signal generated by MGC3130, in this case of frequency 115 kHz and amplitude 2.84 V. Figure 12b shows the signal received from one of the Rx electrodes when there is no object on the sensitive area modifying the electrical field. As expected, the frequency is 115 kHz, but the amplitude of the received signal is 1.82 V. Figure 12c shows the same Rx signal, but introducing an object in the sensitive area. The amplitude of the signal varies up to 2.18 V.

### 2.3. Textile 3D Gesture Sensor Design

Starting from the design data for the sensitive electrodes from Microchip, several of our own designs have been developed based on textile substrates. A similar design to Microchip PCB has been made but using the textile substrate as the dielectric layer.

The manufacturing technology used to implement this type of sensor was based on serigraphic technology of thick film. The screen-printing process consists of forcing pastes of different characteristics over a substrate through some screens using squeegees. Openings in the screen define the pattern that will be printed on the substrate by serigraphy. The final thickness of the pastes can be adjusted by varying the thickness of the screens. In the same way as for a PCB manufacturing, conductive materials and dielectric materials have been employed. Conductive silver ink has been employed to make the electrodes. In the case of the dielectrics, textile, dielectric inks, and polyurethane plastic films have been the materials used.

Different types of textiles have been selected in function of the material used for their manufacturing. The aim is to study their relative permittivity as well as their thickness. Both parameters influence in the value of the associated capacitances as analysed previously.

For each design, type of textile, and inks, measures of their physical and electrical parameters have been taken. The C_TxRx_, C_RxGND_ and C_TxGND_ capacitances have been measured as well.

**Figure 13:**
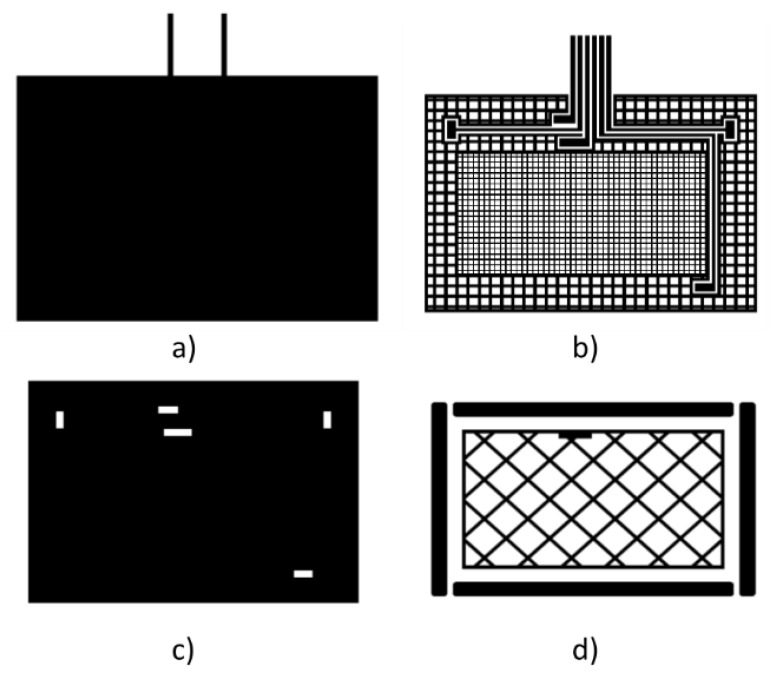
3DS-1 design with four layers: (**a**) ground plane layer, (**b**) transmission Tx electrode, (**c**) dielectric layer between Rx and Tx layers and vias, (**d**) Rx electrode layer.

The first design made, named 3DS-1, is shown in Figure 13. It consists of a ground plane layer (Figure 13a), a layer containing the Tx electrode and the connection lines of Rx with MGC3130 (Figure 13b), a dielectric layer provided with vias that allow the connection between the Rx layer and the Rx connection lines (Figure 13c), and the Rx electrode layer with 4 perimeter electrodes and one central (Figure 13d). The dimensions of the sensor are identical to the 95 × 60 sensor from Microchip explained previously. In this design, the connection lines of the Rx electrodes have been placed on the transmit layer to minimize interferences.

**Figure 14:**
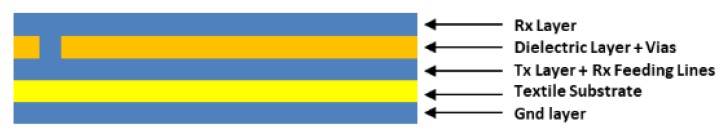
Cross-section of the **3DS-1** sensor. In addition to the 4 layers shown in Figure 13 the textile substrate between the ground plane layer and the Tx electrode layer can be observed.

Figure 14 shows the cross-section of the sensor. The complete structure contains 5 layers since the textile substrate is used as a dielectric layer between the ground plane layer and the Tx electrode layer. The different layers, except the substrate, are screen-printed. Silver ink was employed for the conductive materials. Dielectric inks were used in the case of the dielectric layer between the Rx electrode layer and the Tx electrode layer. With this design, high capacitances are obtained out of the range recommended by Microchip for use with MGC3130. In part, this is due to the solution employed for the dielectric with worse performance than expected. This will be discussed in the Results Section.

**Figure 15:**
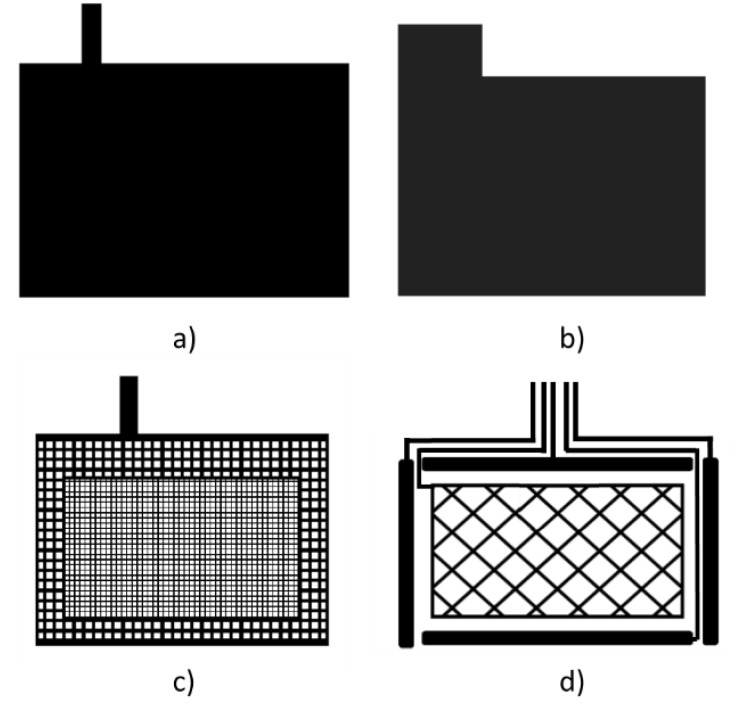
3DS-2 design with four layers. Ground plane layer (**a**). Dielectric layer between Tx and ground layer (**b**). Transmission Tx layer (**c**). Rx layer (**d**).

The second design made, named 3DS-2, is shown in Figure 15. It consists of a ground plane layer (Figure 15a), a dielectric layer between the ground plane layer and the Tx layer (Figure 15b), a layer containing the transmission Tx electrode Figure 15c) and a layer with the reception Rx electrodes (Figure 15d). The textile substrate acts as a dielectric between Rx and Tx layers. In this case, making the vias on the textile substrate is not viable. Hence, the connection lines between the Rx electrodes and MGC3130 have been made on the same Rx layer. This design allows us to decrease the C_TxRx_ and C_RxGND_ capacitances since it is possible to utilize a textile substrate with a high thickness.

**Figure 16:**
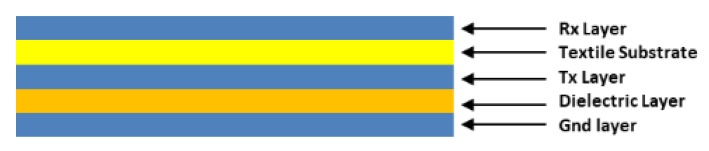
Cross-section of the 3DS-2 sensor. In addition to the 4 layers shown in Figure 15, the textile substrate between the Rx layer and the Tx electrode layer can be observed.

Figure 16 shows the cross-section of the sensor. The complete structure contains 5 layers since the textile substrate is used as a dielectric layer between the Rx electrode layer and the Tx electrode layer. The different layers, except the substrate, are screen-printed employing silver ink for the conductive materials and dielectric inks in the case of the dielectric layer between the Rx electrode layer and the Tx electrode layer. The main problem of this design comes from the alignment of the connection lines. The use of a textile substrate makes it difficult for the correct alignment of the Rx and Tx layers. For this reason, a final design with a different connector position was proposed to solve this problem. The design is shown in Figure 16.

Both designs have been developed with 8 different types of textiles (Table 4 and Table 5). The aim is to determine the influence of the thickness and the relative permittivity of the textiles on the sensor. Each one of the employed textiles have been characterized physically and electrically.

Regarding the inks, one conductive silver ink (Table 6) and three types of dielectric inks have been employed (Table 7). The inks influence noticeably the final capacitance at a thickness level as well as at a relative permittivity level. Heat sealed polyurethanes (Table 8) have been employed as well in some sensors as substitutes of the dielectric inks.

Once the different sensors were built, they have been characterized electrically by means of the determination of their C_TxRx_, C_RxGND_, and C_TxGND_ capacitances. Lastly, the sensitivity of the different sensors has been determined using a graphical user interface software from Microchip, AUREA.

## 3. Materials and Methods

### 3.1. Materials

Different cotton, polyester and mixed fabrics with different fabric densities, yarn diameter, and weave have been used. Table 4 and Table 5 show the main characteristics of the fabrics used.

The conductive ink (Table 6) used is silver IPC-603X from INKRON (Kutojantie, Espoo, Finland).

Three dielectric inks (Table 7) with different flexibility and stretchability characteristics have been used. The inks are 127-48D from CREATIVE (Lamesa, Texas, USA), DI-7542 from EMS (Delaware, Ohio, USA) and IPD-670 from INKRON.

Two polyurethane films (Table 8) have been used. The films are EU94DS from DELSTARD and UAF445 from ADHESIVE FILMS.

### 3.2. Sensor Development

Manufacturing technology used was based on serigraphic technology of thick film. The screen-printing process consists of forcing pastes of different characteristics over a substrate through some screens using squeegees. Openings in the screen define the pattern that will be printed on the substrate by serigraphy. The final thickness of the pastes can be adjusted by varying the thickness of the screens. When screen-printing technology is used, it is necessary to manufacture frames with screen mesh for the design.

The screen for the conductors was a 230 mesh polyester material (PET 1500 90/230-48 from Sefar) and the screen for dielectric layer was a 76 mesh polyester material (PET 1500 30/76-120 PW from Sefar). Afterwards, to transfer the pattern to screen mesh, a UV film Dirasol 132 from Fujifilm was used. The final screen thickness was 74 μm for the screen for conductors and 217 μm for the screen for the dielectric. The patterns were transferred to the screen by using a UV light source unit IC-5000 from BCB.

Printing was carried out by using E2XL from EKRA screen-printer with a shore 75° hardness squeegee, 60° squeegee angle, 1 mm snap-off, 3.5 bar force, and 100 mm/s.

After the deposition of the inks, these were cured in an air oven FED-115 from BINDER at 130 °C for 15 min in order to use the same curing characteristics for all inks. In the case of DI-7542 from EMS a UV oven Ncure-Lab/Static 120 from EneMaq was used with 0.5 J/cm^2^.

The polyurethanes have been heat sealed on the fabrics with a DCH-100 heatpress from Microtec at 130 °C for 60 s.

### 3.3. Measurements

The capacitance between electrodes was measured at 100 kHz with a KEYSIGHT U1733C LCR meter.

The relative permittivity (*ε_r_*) measures were carried out with a Hewlett Packard 4263A LCR meter. The following measurement accessories were used: Hewlett Packard 16089B Kelvin Clips Leads and a Yokogama-Hewlett Packard 16451A Dielectric Test Adaptor. The LCR meter was configured to measure a tension level of 1V, with an average of 64 samples and a low read rate (Level = 1 V, Avg = 64, Meas Time= Low). The measurement mode was Cp and D (parallel capacity and loss tangent). The capacity measurements were taken at three different parts of the fabric with a 4-frequency scan (0.1 kHz, 1 kHz, 10 kHz, and 100 kHz). The *ε_r_* value was obtained directly from the Cp value.

The measurements of the thickness of the fabric were taken at 4 different points using a Mitutoyo CP calibre CD-6” with a 10 µm resolution.

Macroscopic images were taken with a LEICA MZ APO stereomicroscope.

## 4. Results and Discussion

**Figure 17:**
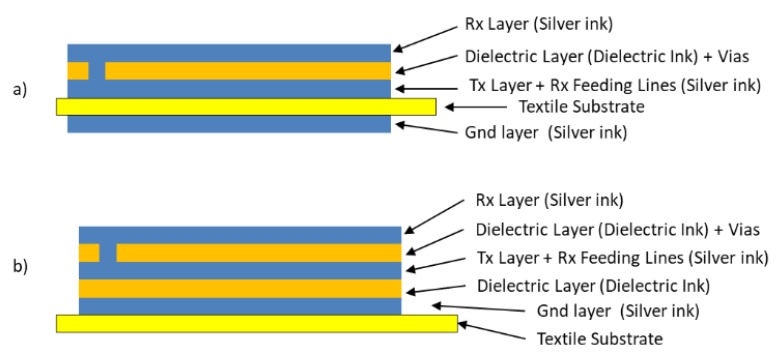
3DS-1 Design with two construction structures: (**a**) the textile substrate acting as a dielectric between the Tx electrode and the ground plane (sensor name **3DS_1a**) and (**b**) the textile substrate acting as a mere base (sensor name **3DS_1b**).

**Figure 18:**
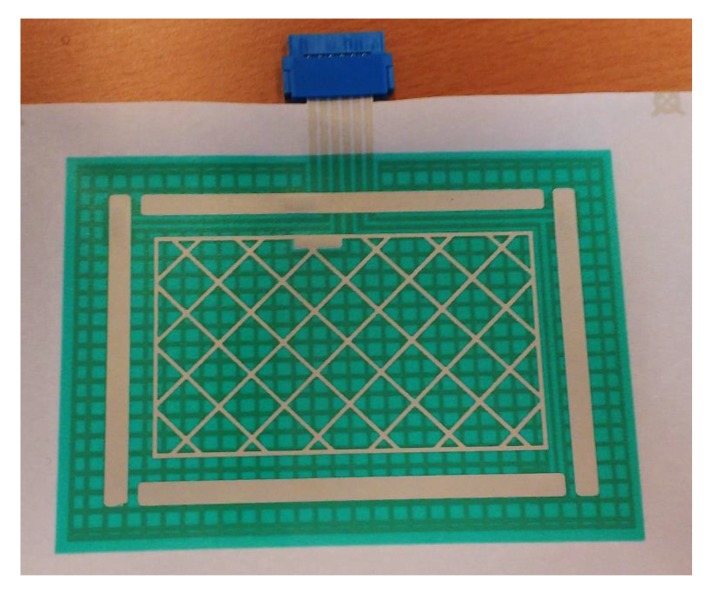
3DS-1 Sensor.

In the 3DS-1 design, the dielectric layer between the Rx electrodes and the Tx electrode must mandatorily be a screen-printed layer of dielectric ink, since this layer contains the connection traces. Implementing these traces with vias in a layer made with textile is practically impossible. Thus, in this design, the substrate textile can be used as the dielectric layer between Tx layer and GND layer (Figure 17a). The textile provides its physical and electrical characteristics, or it can only be utilized as the base substrate (Figure 17b) where it only contributes with its physical characteristics. Figure 18 shows the final sensor, the appearance being the same in both of the implemented structures.

As aforementioned, the C_RxTx_, C_TxG_, and C_RxG_ capacitances must be of a low value and within a very concrete range. Once the dimensions of the areas of these capacitors are fixed, there are only two parameters to be modified to achieve a low capacitance. These parameters are the layer thickness and the relative permittivity. For this reason, the three selected dielectrics have been characterized looking for the one with the lowest relative permittivity. Regarding the layer thickness, a large thickness would be desirable. To increment the thickness, the physical parameters of the screen-print must be varied. The mesh must be low in the screen to augment the quantity of ink to be printed and several layers must be screen-printed successively to increase the thickness. Table 9 shows the relative permittivities of the three available inks, where CREATIVE 12-48D presents the lowest relative permittivity. The right column shows the minimum thickness recommended by Microchip to achieve an appropriate value of C_RxTx_. These large values of thicknesses cannot be obtained using screen-print techniques.

A first prototype with type A substrate was implemented to characterize the sensor. Two sensors were implemented: a sensor named 3DS-1a-TA and another one named 3DS-1b-TA. To be able to estimate the capacitances using theoretical calculus, the value of the relative permittivity of the textiles is needed. Table 10 shows the determined values. The right column shows the minimum thickness recommended by Microchip to achieve an appropriate value of C_RxTx_, considering the permittivity obtained from the textiles. Type A textile is located well below the limit; Type C textile is located below as well, and the rest are on the limit or exceed it widely.

Table 11 shows the different values obtained from 3DS-1a-TA for C_TxRx_, C_RxGND_, and C_TxGND_. The average dielectric thickness obtained between the Rx and Tx layers is 20 μm. Type A textile has an average thickness of 110 μm. The average thickness between Rx and GND is 135 μm.

Table 12 shows the different values obtained from 3DS-1b-TA for C_TxRx_, C_RxGND_, and C_TxGND_. The average dielectric thickness obtained between Rx and Tx layers is 40 μm. The average thickness between Tx layer and GND is 60 μm. The average thickness between Rx layer and GND is 100 μm.

**Figure 19:**
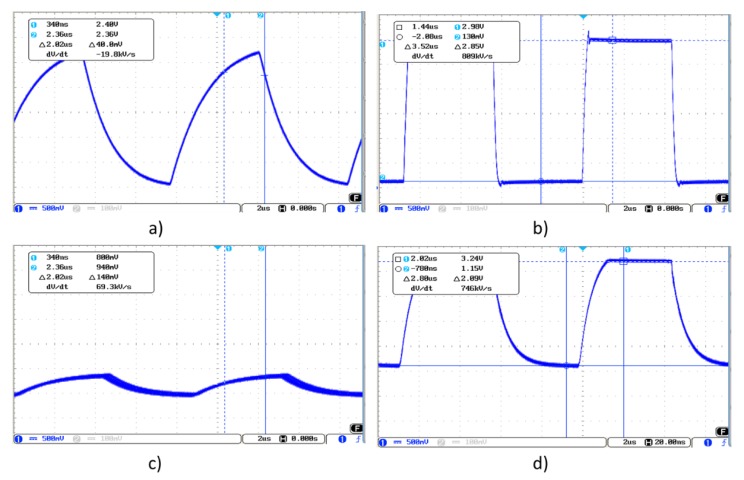
Transmission signal waveform (**a**), a signal deformation can be observed due to a capacitance of CTxGND > 1 nF. Regenerated signal obtained coupling an AO between the Tx pin and the Tx electrode (**b**). Receiving Rx signal with direct connection (**c**) between the Tx pin and the Tx electrode. Receiving Rx signal with AO (**d**) between the Tx pin and the Tx electrode.

The capacitances obtained with the 3DS-1a-TA sensor as well as with 3DS-1b-TA, widely exceed the margins recommended by Microchip. This is fundamentally due to the fact that the reached thicknesses with the screen-printed dielectric layers do not achieve the minimum values recommended by Microchip. These high capacitance values affect the input and output signals from/to the controller. Figure 19 shows the waveforms from the 3DS-1a-TA sensor. The transmission signal (Figure 19a) presents a deformation due to a capacitance of C_TxGND_ > 1 nF. If a buffer operational amplifier is coupled between the Tx pin and the Tx electrode, a regenerated signal is obtained (Figure 19b). Figure 19c shows the receiving Rx signal with direct connection between the Tx pin and the Tx electrode. Figure 19d shows the receiving Rx signal with an AO between the Tx pin and the Tx electrode. The results obtained with the 3DS-1b-TA sensor are very similar to those obtained with the PCB reference sensor (Figure 12).

These capacitance values out of the recommended range have forced the use of the 3DS-2 sensor. This sensor allows us to modify the thickness as well as the relative permittivity of the dielectric Rx-Tx since the textile substrate is used as dielectric (Figure 16). When studying the response using the same textile substrate (Type A) but in 3DS-2 configuration, a significant reduction of the capacitance values is observed (Table 13), although the values are still out of the range recommended by Microchip. This high value out of the range comes from the yet insufficient dielectric thickness, since the type A textile has a thickness of 110 μm. For this reason, textiles with a noticeably higher thicknesses have been employed, between 380 and 1300 μm. In this design, the connection lines of the Rx electrodes with the connector are located on the same layer as the Rx electrodes. Thus, the lines influence the total capacitance. In the case of the North electrode, the connection line is out of the sensitive area and has least influence, getting a lower capacitance with respect to the rest.

**Figure 20:**
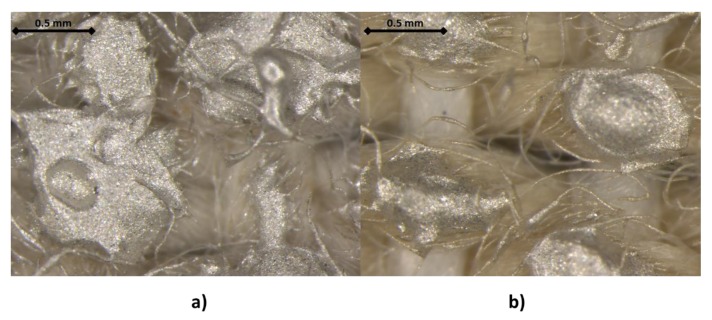
25× Magnified view of the printing of the conductive ink on the Type D textile (**a**) and on the Type E textile (**b**).

The rest of the textiles were fabrics with large thicknesses and presented much roughness. This led to problems with the printing of the conductive and dielectric inks. A couple of samples are presented in Figure 20. On the left, a printing on a type D textile can be observed, and, on the right, a printing on a type E textile. In both textiles, the conductive ink is not uniformly distributed on the textile, leading to very high resistance values, or even infinite, i.e., open circuits or with no electrical continuity. This problem appears on Type B, C, D, E, and F textiles, but on the contrary, in Type G, H, and I, it is possible to screen print the conductive silver ink with no errors of continuity.

**Figure 21:**
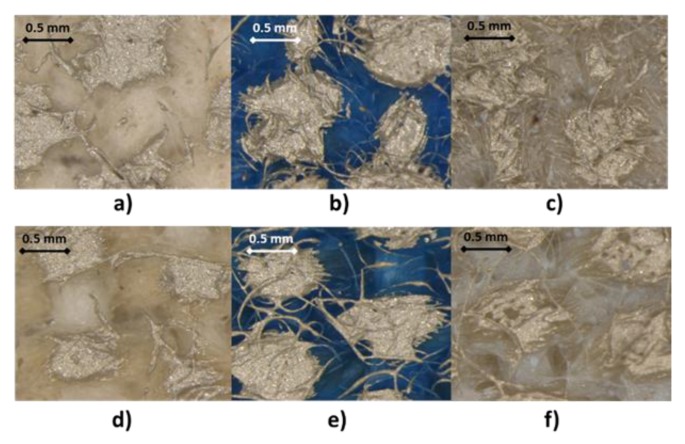
25× Magnified view of the printing of the dielectric and conductive inks on: (**a**) Type D textile with dielectric Creative 127-48D and a layer of silver ink, (**b**) Type D textile with dielectric EMS DI-7542 and a layer of silver ink,(**c**) Type D textile with dielectric Inkron IPD-670 and a layer of silver ink, (**d**) Type E textile with dielectric Creative 127-48D and a layer of silver ink, (**e**) Type E textile with dielectric EMS DI-7542 and a layer of silver ink and (**f**) Type D textile with dielectric IPD-670 and a layer of silver ink.

This problem can be solved modifying the surface of the textile to soften the roughness. The modification consists of the printing of a dielectric layer on the textile or the heat sealing of a polyurethane film. In the case of the printing of a dielectric layer, the three available dielectrics have been used on each one of the textiles. Later, a conductive silver ink layer was printed to check if there is any improvement in the result of the printing. Figure 21 shows the result of the printing of the different dielectric inks: (a) Type D textile with dielectric Creative 127-48D and a layer of silver ink, (b) Type D textile with dielectric EMS DI-7542 and a layer of silver ink, (c) Type D textile with dielectric Inkron IPD-670 and a layer of silver ink, (d) Type E textile with dielectric Creative 127-48D and a layer of silver ink, (e) Type E textile with dielectric EMS DI-7542 and a layer of silver ink and (f) Type D textile with dielectric IPD-670 and a layer of silver ink.

The problem of the lack of continuity in the conductive tracks could not be solved with the dielectrics. This led to the use of heat sealable polyurethanes on the different textiles. Specifically, EU94DS from Delstar Inc., and UAF-445 from Adhesive Films. These polyurethanes (Table 8) can help to improve the capacities of the sensor providing a larger layer thickness (80 μm in the case of EU94DS and 120 μm the case of UAF-445). In addition, they can improve the relative permittivity. Table 14 shows the permittivities measured for each one of the used polyurethanes. The printing of the conductive ink on the textiles covered with polyurethanes is perfect.

**Figure 22:**
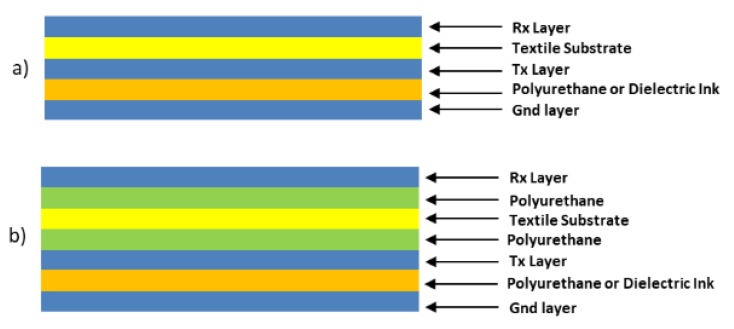
3DS-2 Design with two construction structures: (**a**) the textile substrate acting as a dielectric between the Tx electrode and Rx electrode (sensor named 3SD_2a) and (**b**) the textile substrate covered with polyurethane (sensor named 3SD_2b).

With the employment of the polyurethanes, the structure of the 3DS-2 sensor changes depending on the textiles. For Type G, H, and I textiles, the structure of Figure 22a (named 3SD_2a) has been employed and for Type B, C, D, E, and F textiles, the structure of Figure 22b (named 3SD_2b) has been employed.

**Figure 23:**
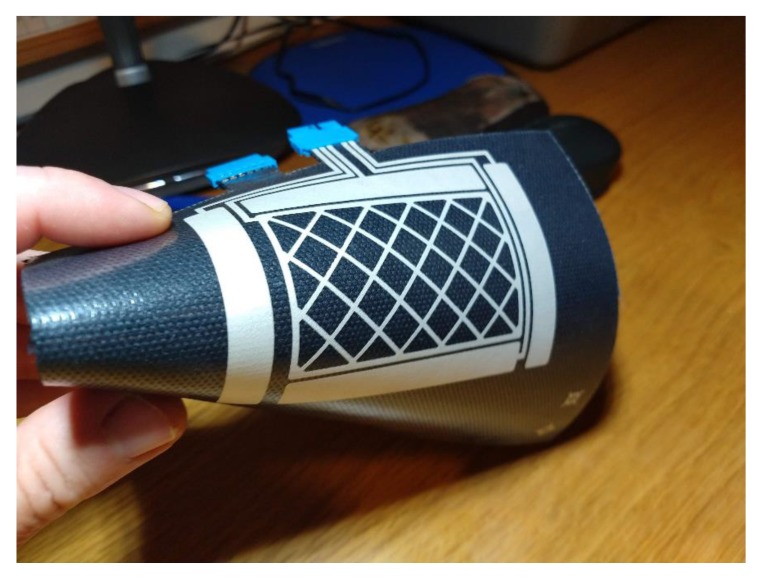
Sensor 3DS-2a.

Figure 23 shows the final aspect of one of the sensors made, concretely the 3DS-2a sensor with Type I textile (named 3DS-2a-TI).

Table 15 shows the values of the capacitances associated to each one of the types of developed sensors. A remarkable decrease of the capacitance values can be noticed. This is due to the increase of the thickness of the substrates and the values of the relative permittivities. The sensor that most met expectations was 3DS-2a-TI with very low values of C_TxRx_ and C_RxGND_ capacitances, far below the recommended limit by Microchip. Regarding the value of the C_TxGND_ capacitance, it is situated above the recommended value (<1 nF). It can be solved inserting an op-amp between the Tx pin and the Tx electrode as recommended by Microchip.

**Figure 24:**
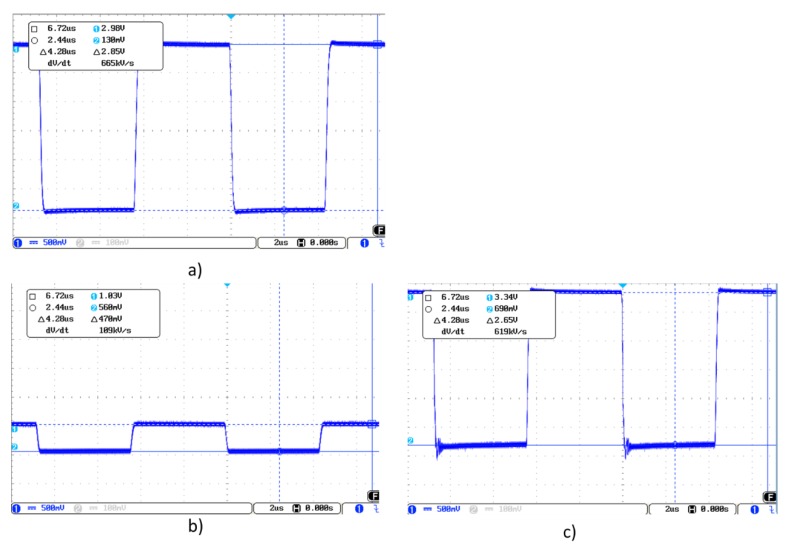
Waveform of the transmission signal (**a**) with buffer due to the capacitance C_TxGND_ > 1 nF, (**b**) receiving RX signal with direct connection between the Tx pin and the Tx electrode and (**c**) receiving RX signal with op-amp between the Tx pin and the Tx electrode.

Figure 24 shows the waveform of the 3DS-2a-TI sensor. Figure 24a shows the Tx signal with a buffer OA inserted between the Tx pin and the Tx electrode due to the value of C_TxGND_, greater than 1 nF. Figure 24b shows the signal in one of the Rx electrodes when there is no object modifying the electrical field. The Tx signal was the original with no buffer. Figure 24c shows the same Rx signal after inserting the buffer in the path of the Tx signal.

**Figure 25:**
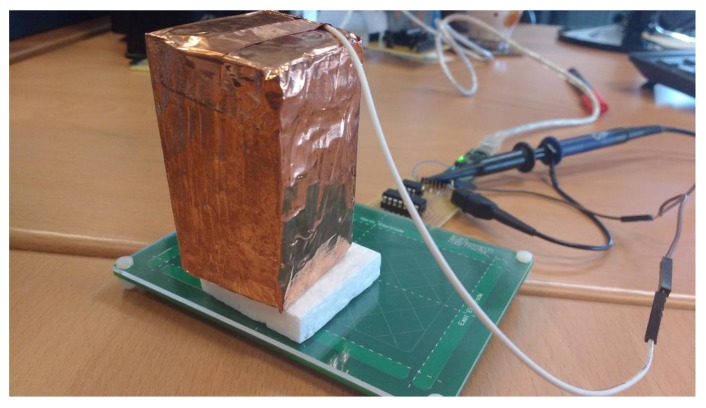
“Artificial hand” provided by Microchip. It is made of styrofoam covered by copper and connected to ground. Some blocks of styrofoam with no covering allow to move the “artificial hand” away from the sensor.

**Figure 26:**
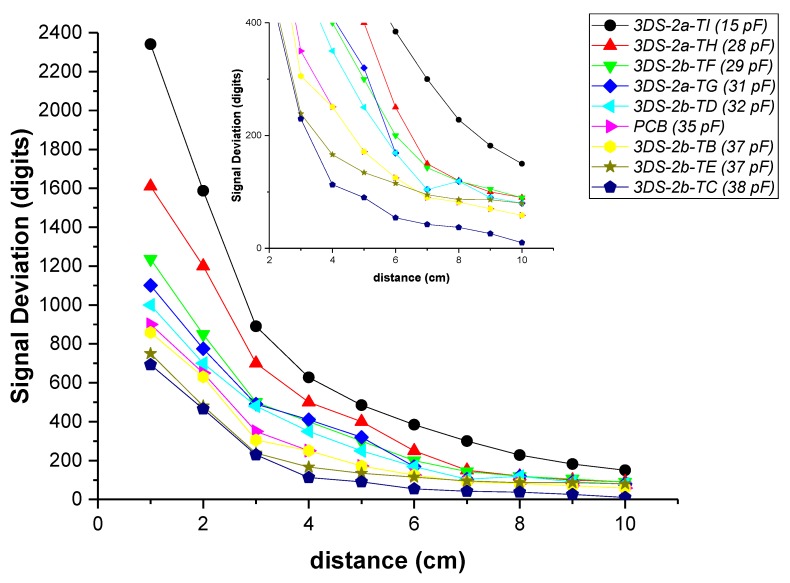
Signal Deviation of the different sensors in function of the distance of the hand from the surface of the sensor. The C_TxRxN_ value, in brackets in the legend, has been included as a reference to assess the relationship between capacitance and sensitivity. This relationship is the same in any of the associated capacities.

Microchip provides the AUREA graphical user interface that allows for characterizing the sensors. The sensitivity is, maybe, the most important parameter of these sensors. Microchip [43] provides an “artificial hand”, a 40 × 40 × 70 mm styrofoam (*ε_r_* ≈ 1) cube covered with an adhesive copper sheet. This block must be connected to ground to simulate the conditions of the human body. To determine the sensitivity, the block is placed at different distances from the surface of the sensor using blocks of styrofoam of different thicknesses (1, 2, … cm) (Figure 25). AUREA allows us to read the obtained data. Figure 26 shows a representation of the data. It can be observed that as the associated capacitances increase, the signal deviation decreases for the same distance to the surface. The 3DS-2a-TI sensor presents the best sensitivity. The sensitivity is even better than the one of the PCB’s sensor from Microchip, due to the low value of their associated capacitances.

It would be convenient to be able to determine the value of the associated sensor capacities prior to their manufacturing. Thus, only those textiles that achieve capacities below 30 pF would be utilized in the sensors.

Two theoretical values have been calculated, the nominal theoretical value (C_n_) calculated with Equation (1) and the edge effect capacitance value (C_edge_). This latter value considers the effect of the field lines around the edges of the capacitor and can be calculated according to Equations (2) and (3).

(1) $$C=\varepsilon_{r}\cdot \varepsilon_{0}\frac{L\cdot w}{t}$$

(2) $$C=\frac{\varepsilon_{0}\cdot \varepsilon_{r}\cdot \left(L+\Delta f\right)\cdot \left(w+\Delta f\right)}{t}$$

(3) $$\Delta f=t+\frac{\varepsilon_{0}\cdot t\cdot 10\cdot \ln\left(\left(L+w\right)+1\right)}{\pi }$$

where *C* is the value of the capacitance in pF, *L* is the length in cm, *w* is the width in cm, *t* is the thickness in cm, *ε_r_* is the relative permittivity, and *ε*_0_ is the vacuum permittivity (8.85 × 10^−12^ F/m).

A comparative study has been made with the North electrode. This electrode is the one with less influence of the capacitances associated to the Rx conduction lines to the connector. Table 16 shows the values of the theoretical capacities and the real C_TxRx_ value measured with an LCR meter. In general, the theoretical results are validated by the real ones. The C_edge_ value is very close to the real one in all the cases. Thus, it is possible, knowing the relative permittivity of each textile and its thickness, to deduct the value of its associated capacitances prior to the sensor manufacturing.

**Figure 27:**
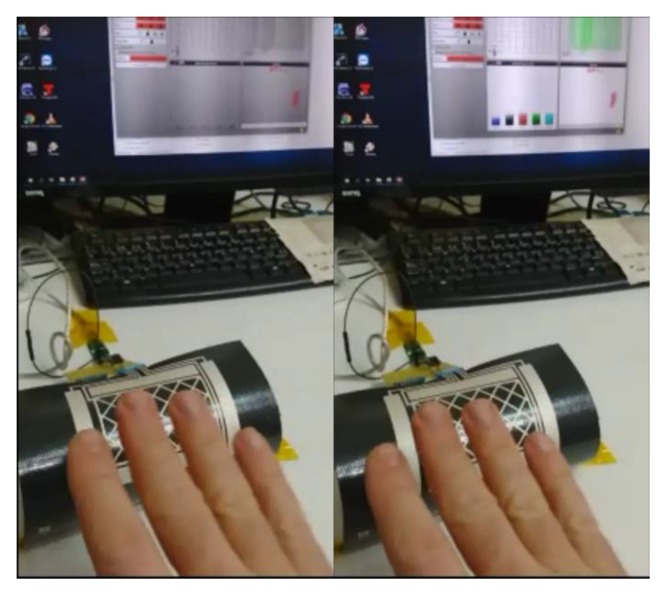
Approach detection.

**Figure 28:**
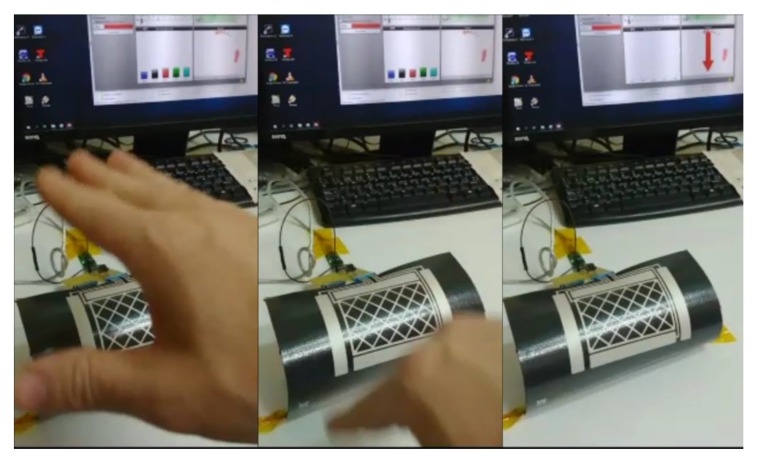
Flick north to south.

**Figure 29:**
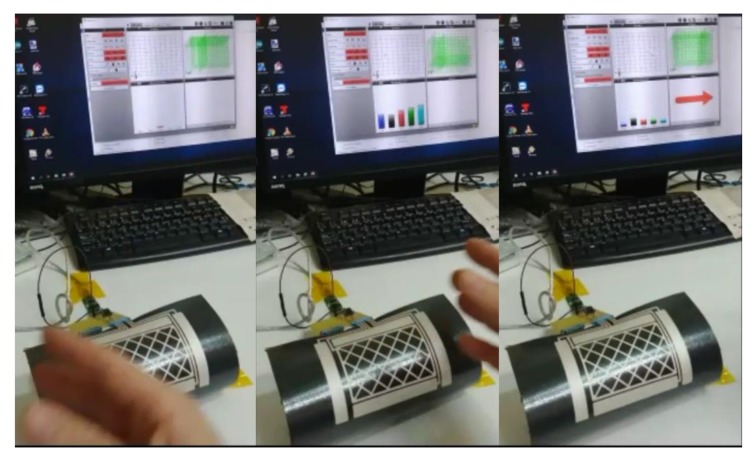
Flick west to east.

**Figure 30:**
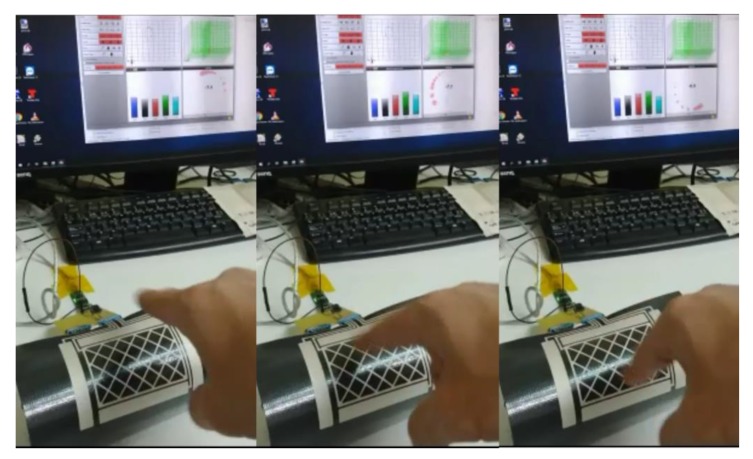
Airwheel.

The following figures show the frames of a video with some gestures that the controller can recognize: Figure 27. Approach detection.; Figure 28. Flick north to south.; Figure 29. Flick west to east.; and Figure 30. Airwheel. The videos are available as Supplementary Materials (Videos S1 and S2).

**Figure 31:**
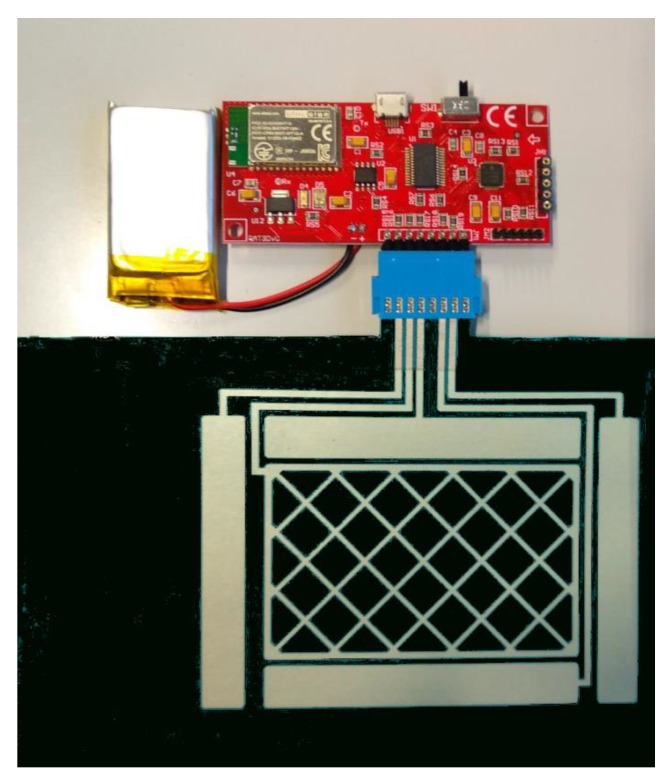
Complete portable system.

**Figure 32:**
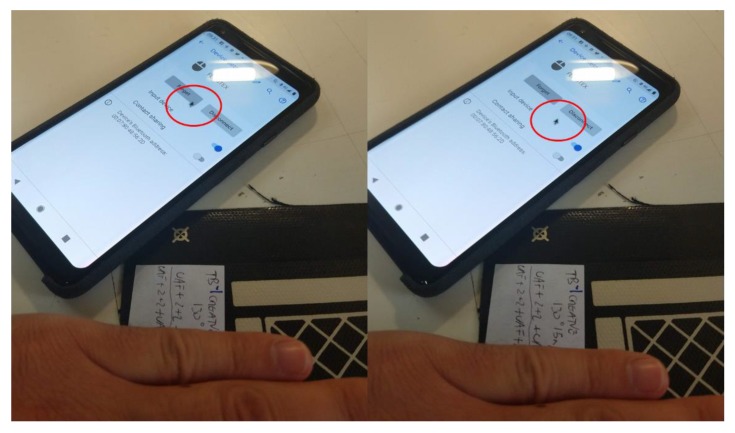
3D Sensor used as a Wireless mouse with a mobile phone.

A Bluetooth portable system has been developed to be used with mobile devices (Figure 31). The system is configured with the AUREA application and the configuration parameters are saved in the portable device. To check the functioning, it is connected to an Android device as wireless mouse as shown in Figure 32.

## 5. Conclusions

The aim of this research was to obtain a wearable textile 3D gesture recognition sensor based on the Microchip 3D GestIC^®®^ sensor design. The fundamental parameters of that sensor design are the capacitance between transmission and reception electrodes. The value of this capacitance depends fundamentally on the thickness of the substrate and its relative permittivity. Therefore, the key to the design is to look for the best textile substrate materials. But the best textile material is not always the best material to be used with screen-printed technology. For this reason, a large proportion of the research has consisted of trying different fabrics. Fabrics are composed of different materials and, therefore, with different thickness and relative permittivity but also with different behaviour regarding silver and dielectrics inks. A flexible textile 3D sensor was developed after studying all these parameters.

That sensor was characterized in relation to its electrical parameters, namely its capacitances between electrodes, its thickness, and its sensitivity to the presence of a hand. The characteristics obtained were better than Microchip’s PCB design and with the further advantage of its flexibility. The Microchip driver (MGC3XXX) incorporates a calibration procedure that can be programmed, for example, on stand-by periods. This allows the driver to adjust to the base signal according to the bending angle. This feature maximizes the advantage of using a flexible sensor.

We are currently working on the study of the variation of two important features of textiles, such as softness or permeability. The use of soft polyurethane films guarantees softness. Regarding the permeability, the addition of different layers reduces it. We are currently exploring different solutions such as microperforations and hole patterns. Lastly, we are preparing some experiments to determine the washing capacity of the sensor, due to the importance of washing in the textile area.

In conclusion, a 3D gesture sensor based on E-field change technologies has been developed to be used with textile substrates using a low cost and common textile industry printing technique: screen-printing. The system works on both flat and curved surfaces, which allows it to be used in several areas: clothes, automobile industry, healthcare, etc.

## Figures and Tables

**Table 1:** Values of C_TxRx_ [5–30 pF].

	l (mm)	w (mm)	t (mm)	Microchip (pF)	Value (pF)
R_xN_	91.7	5.0	0.935	20.00	33.92 ± 10.67
R_xS_	91.7	5.0	0.935	20.00	34.66 ± 10.69
R_xE_	70.5	5.0	0.935	18.00	30.09 ± 10.60
R_xW_	70.5	5.0	0.935	18.00	30.62 ± 10.61
R_xC_	85.7	50.5	0.935	65.00	68.22 ± 11.36

**Table 2:** Real values of CTxGND [<1 nF].

	l (mm)	w (mm)	t (mm)	Microchip (pF)	Value (pF)
Tx	120	85	0.540	590.00	635.00 ± 22.70

**Table 3:** Values of CRxGND [5–30 pF].

	l (mm)	w (mm)	t (mm)	Value (pF)
R_xN_	91.7	5	0.1512	34.31 ± 10.68
R_xS_	91.7	5	0.1512	33.19 ± 10.66
R_xE_	70.5	5	0.1512	30.30 ± 10.60
R_xW_	70.5	5	0.1512	30.17 ± 10.60
R_xC_	120.0	85	0.1512	62.85 ± 11.25

**Table 4:** Fabric characteristics (I): composition and ligament.

Fabric	Picture	Weft Material	Warp Material	Ligament	
Type A 100% Polyester	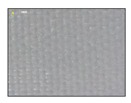	Polyester	Polyester	Taffeta	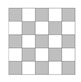
Type B 50% Cotton 50% Polyester	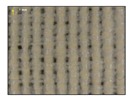	Cotton	Polyester	Taffeta	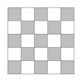
Type C 100% Cotton	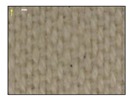	Cotton	Cotton	Twill	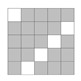
Type D 100 % Cotton	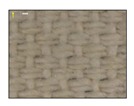	Cotton	Cotton	Teleton	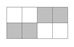
Type E 100% Polyester	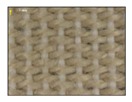	Polyester	Polyester	Taffeta	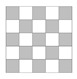
Type F 100 % Cotton	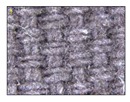	Cotton	Cotton	Teleton	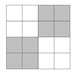
Type G Polyurethane	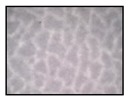	Polyurethane	Polyurethane	Non-woven	
Type H 100% Cotton	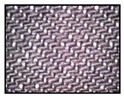	Cotton	Cotton	Twill	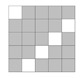
Type I Polyurethane	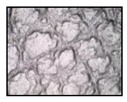	Polyurethane	Polyurethane	Non-woven	

**Table 5:** Fabric characteristics (II): size and weight characteristics.

Fabric	Weft Density (Thread/cm)	Warp Density (Thread/cm)	Fabric Density (Thread/cm^2^)	Wire Weft Diameter (µm)	Wire Warp Diameter (µm)	Thickness (µm)	Grammage (g/m^2^)
Type A	24	38	62	300	300	110 ± 08	112 ± 4
Type B	13	26	39	450	450	380 ± 07	181 ± 1
Type C	26	34	60	300	300	470 ± 20	235 ± 2
Type D	10	28	38	400	400	530 ± 10	312 ± 5
Type E	10	22	32	350	350	570 ± 11	226 ± 4
Type F	7	24	31	450	450	700 ± 19	324 ± 2
Type G	-	-	-	-	-	720 ± 15	75 ± 1
Type H	20	20	40	360	360	920 ± 11	105 ± 3
Type I	-	-	-	-	-	1300 ± 16	152 ± 5

**Table 6:** Silver ink characteristics.

	INKRON IPC-603X
Sheet Resistivity (mΩ/sq/mil)	<15
Solids (%)	100
Viscosity (Pas)	16 @0.25 s^−1^
Curing	130 °C–15 min
Properties	● High Stretchability ● Flexible

**Table 7:** Dielectric ink characteristics.

	CREATIVE 127-48D	EMS DI-7542	INKRON IPD-670
Viscosity (Pas)	15–20	7 @0.05 s^−1^	32 @2.5 s^−1^
Screens polyester [threads/inch]		156–305	
Curing	125 °C–60 min	0.5 J/cm^2^	130 °C–15 min
Properties	● Flexible	● Flexible ● UV-Cure	● Stretchable

**Table 8:** Polyurethane characteristics.

	DELSTAR EU94DS	ADHESIVE FIMS UAF-445
Thickness (µm)	80	120
Weight (g/m^3^)	94	-
MVTR * upright (g/m^2^/24 h) @37 °C	400	-
Tensile Strength MD ** (gf/cm)	3000	-
Elongation at break MD ** (%)	700	450

* Moisture vapor transmission rate (MVTR), ** Machine direction (MD).

**Table 9:** Relative permittivity of the dielectric inks *ε_r_* @100 kHz.

Dielectric	Relative Permittivity	t > *ε_r_*/5 (μm)
CREATIVE 127-48D	1.72	344
EMS DI-7542	5.68	1136
INKRON IPD-670	4.20	840

**Table 10:** Relative permittivity of fabrics *ε_r_* @100 kHz.

Fabric	Relative Permittivity	Thickness (µm)	t > *ε_r_*/5 (μm)
Type A	2.37	110 ± 8	474
Type B	1.93	380 ± 7	386
Type C	2.58	470 ± 20	516
Type D	2.64	530 ± 10	528
Type E	1.37	570 ± 11	274
Type F	2.65	700 ± 19	530
Type G	1.42	720 ± 15	284
Type H	3.41	920 ± 11	680
Type I	1.64	1300 ± 16	328

**Table 11:** Capacitance values of 3DS-1a-TA (pF).

C_TxRxN_	229.6 ± 14.6
C_TxRxS_	261.9 ± 15.2
C_TxRxE_	210.1 ± 14.2
C_TxRxW_	254.3 ± 15.1
C_TxRxC_	554.9 ± 21.1
C_RxNGND_	219.7 ± 14.4
C_RxSGND_	258.8 ± 15.2
C_RxEGND_	202.8 ± 14.0
C_RxWGND_	242.4 ± 14.8
C_RxCGND_	449.6 ± 19.9
C_TxGND_	1990.0 ± 13.9

**Table 12:** Capacitance values of 3DS-1b-TA (pF).

C_TxRxN_	301.0 ± 16.0
C_TxRxS_	354.6 ± 17.1
C_TxRxE_	276.0 ± 15.5
C_TxRxW_	326.2 ± 16.5
C_TxRxC_	716.2 ± 24.3
C_RxNGND_	296.4 ± 15.9
C_RxSGND_	357.2 ± 17.1
C_RxEGND_	274.1 ± 15.5
C_RxWGND_	317.9 ± 16.3
C_RxCGND_	671.8 ± 23.4
C_TxGND_	3430.0 ± 16.9

**Table 13:** Capacitance values of 3DS-2-TA (pF).

C_TxRxN_	97.2 ± 11.9
C_TxRxS_	132.4 ± 12.6
C_TxRxE_	114.5 ± 12.2
C_TxRxW_	110.9 ± 12.2
C_TxRxC_	188.1 ± 13.7
C_RxNGND_	95.1 ± 11.9
C_RxSGND_	129.0 ± 12.5
C_RxEGND_	111.6 ± 12.2
C_RxWGND_	108.5 ± 12.1
C_RxCGND_	178.6 ± 13.5
C_TxGND_	1915.0 ± 48.3

**Table 14:** Relative permittivity of the polyurethanes.

	DELSTAR EU94DS	ADHESIVE FILMS UAF-445
*ε_r_* @100 kHz	1.46	1.86

**Table 15:** Capacitance values of 3DS-2a and 3DS-2b (pF).

	3DS-2b-TB	3DS-2b-TC	3DS-2b-TD	3DS-2b-TE	3DS-2b-TF	3DS-2a-TG	3DS-2a-TH	3DS-2a-TI
C_TxRxN_	37.4 ± 10.7	38.4 ± 10.7	32.1 ± 10.6	37.4 ± 10.7	29.4 ± 10.6	30.8 ± 10.6	28.9 ± 10.6	15.1 ± 10.3
C_TxRxS_	48.1 ± 10.9	46.0 ± 10.9	40.3 ± 10.8	48.2 ± 10.9	37.8 ± 10.7	43.3 ± 10.9	39.7 ± 10.8	19.3 ± 10.4
C_TxRxE_	39.2 ± 10.7	38.4 ± 10.7	32.9 ± 10.6	38.4 ± 10.7	31.5 ± 10.6	34.1 ± 10.7	32.2 ± 10.6	15.1 ± 10.3
C_TxRxW_	39.6 ± 10.7	36.4 ± 10.7	33.1 ± 10.6	41.4 ± 10.8	29.6 ± 10.5	36.7 ± 10.7	29.6 ± 10.6	16.1 ± 10.3
C_TxRxC_	77.6 ± 11.5	76.4 ± 11.5	63.5 ± 11.2	83.5 ± 11.6	62.8 ± 11.2	64.9 ± 11.3	62.1 ± 11.2	34.7 ± 10.7
C_RxNGND_	37.2 ± 10.7	34.4 ± 10.6	30.6 ± 10.6	37.2 ± 10.7	27.2 ± 10.5	30.6 ± 10.6	28.5 ± 10.6	15.1 ± 10.3
C_RxSGND_	47.7 ± 10.9	52.1 ± 11.0	41.2 ± 10.8	45.8 ± 10.9	35.4 ± 10.7	42.8 ± 10.9	36.8 ± 10.7	19.5 ± 10.4
C_RxEGND_	39.0 ± 10.7	44.3 ± 10.8	32.6 ± 10.6	38.0 ± 10.7	29.1 ± 10.5	33.8 ± 10.7	30.1 ± 10.6	15.1 ± 10.3
C_RxWGND_	39.4 ± 10.7	36.4 ± 10.7	32.0 ± 10.6	42.6 ± 10.8	28.6 ± 10.5	36.4 ± 10.7	29.3 ± 10.6	16.1 ± 10.3
C_RxCGND_	76.8 ± 11.5	71.5 ± 11.4	61.4 ± 11.2	80.2 ± 11.6	60.1 ± 11.2	63.8 ± 11.3	61.8 ± 11.2	34.5 ± 10.7
C_TxGND_	2488.1 ± 59.8	2595.2 ± 61.9	1842.2 ± 46.8	2137.0 ± 52.7	2590.0 ± 61.8	1553.1 ± 41.1	2122.0 ± 52.4	2327.0 ± 56.5

**Table 16:** Values of CTxRx: theoretical nominal capacity (Cn), with Edge effect (Cedge) and real values (Creal) (pF).

	C_n_	C_edge_	C_real_
**3DS-2b -TB**	32.4	35.7	37.4 ± 10.7
**3DS-2b -TC**	30.3	33.7	38.4 ± 10.7
**3DS-2b -TD**	25.2	28.7	32.1 ± 10.6
**3DS-2b -TE**	23.0	25.6	37.4 ± 10.7
**3DS-2b -TF**	17.7	20.5	29.4 ± 10.6
**3DS-2a -TG**	27.1	30.7	30.9 ± 10.6
**3DS-2a-TH**	29.3	33.3	28.9 ± 10.6
**3DS-2a -TI**	8.9	10.9	15.1 ± 10.3

## Supplementary Materials

The following are available online at https://www.mdpi.com/1424-8220/19/23/5068/s1, Video S1: Sensor test, Video S2: Application test.
